# Parametric survival analysis of long COVID among hospitalized patients in Zambia: A retrospective cohort study on the time to symptoms resolving

**DOI:** 10.1371/journal.pgph.0004679

**Published:** 2025-11-06

**Authors:** Warren Malambo, Mutale Sampa-Kawana, Duncan Chanda, Sombo Fwoloshi, Patrick Kaonga

**Affiliations:** 1 University of Zambia, School of Public Health, Department of Epidemiology and Biostatistics, Lusaka, Zambia,; 2 University Teaching Hospital Adult and Emergency Hospital, Department of Internal Medicine, Lusaka, Zambia; 3 Ministry of Health, Lusaka, Zambia; PLOS: Public Library of Science, UNITED STATES OF AMERICA

## Abstract

Long COVID refers to the continuation or emergence of new symptoms within three months after acute SARS-CoV-2 infection, lasting for at least two months. Although several studies have described COVID-19 sequelae, gaps remain in understanding the temporal dynamics of symptoms resolution – information crucial for patients management and recovery planning. This study evaluated the resolution of COVID-19–related symptoms over time and associated factors among hospitalized patients in Zambia. We conducted a retrospective cohort study among individuals discharged after COVID-19 hospitalization and attending follow-up care in 13 specialized clinics in Zambia from August-2020 to December-2022. Severe acute COVID-19 was defined as hospitalization requiring supplemental oxygen, ICU admission, and/or treatment with steroids/remdesivir. Time-to-symptoms resolution (i.e., survival time) and changes in underlying hazard rate were our primary and secondary outcomes, respectively. We estimated incidence rates, median survival time (onset-to-resolution), and factors associated with symptom resolution using survival analysis, including hazard ratios (HRs) and changes in the underlying hazard rate over time. Among 823 participants, 616 (84.3%) had severe acute COVID-19 illness; 50.6% were female, and median age was 54 years (IQR: 43–64). Overall, 597 (72.5%) had symptoms resolution at a median 51 person-days (IQR: 34–104). Most participants (59.4%) had baseline comorbidities, and 16.6% had received ≥1 COVID-19 vaccine dose. Symptoms resolved at a rate of 12.2 per 1,000 person-days. Severe acute COVID-19 was associated with slower symptom resolution (adjusted HR: 0.68, 95% CI: 0.50-0.92), while infection during the Omicron-predominant period compared to wild-type was associated with faster resolution (aHR: 2.71; 95% CI: 1.46-5.03). The hazard rate peaked around person-day 20 and declined thereafter, indicating a non-monotonic recovery pattern. COVID-19 symptoms resolved more rapidly during the first month of post-acute infection. Patients with persistent symptoms not resolved within this period may experience prolonged recovery, underscoring the need for targeted follow-up and supportive care.

## Introduction

Long COVID, a post-viral syndrome, may occur after SARS-CoV-2 infection and affect multiple-organ systems. While some individuals may fully recover shortly after a SARS-CoV-2 infection, others experience persistent symptoms that require ongoing management and support. Long COVID presents in a broad spectrum of symptoms but the most common may include fatigue, shortness of breath, headaches, myalgias, cough and cognitive changes [1]. The World Health Organization defines long COVID as the continuation or development of new symptoms three months after the initial SARS-CoV-2 infection, with symptoms lasting for at least two months and with no other explanation [2]. Despite this definition, a lack of consensus on the clinical presentation of long COVID or a diagnostic test or biomarker for long COVID complicates the categorization and analysis of its sequelae.

A framework, however, suggests that the spectrum of long COVID conditions may be delineated as: i) a group of persisting symptoms that interfere with the ability to function at home or at work and are not explained by major organ injury; ii) new onset of major diseases including diabetes mellitus, cardiovascular diseases, stroke, and pulmonary failure; and iii) tissue injury of multiple organs that has the potential to lead to long-term organ dysfunction [3]. This suggests long COVID may encompass a spectrum of conditions ranging from mild but disabling symptoms (without organ damage) to more severe cases involving the development of serious diseases or actual physical damage to organs.

Evidence on long COVID in Africa has steadily increased despite limited access to health-care services by patients, and inadequate diagnostics and clinical data [4]. The prevalence is estimated to range between 2% to 86% [4–8]. It varies between 10–30% in patients with mild COVID-19, 40–70% in acute COVID-19 hospitalized patients and 10–12% among vaccinated cases [9–13]. Associated factors include older age (≥40 years), underlying comorbidity, severe COVID-19 disease, and admission to intensive care [4,14–16]. Studies have further shown that vaccination is associated with a reduced likelihood of severe acute COVID-19 and long COVID [16–18]. Long COVID has impacts on patients’ quality of life, employment, and income due to diminished functional status or protracted path to recovery [19,20].

Although a number of studies in Zambia have reported COVID-19 sequelae, the knowledge gap still exists around the temporal dynamics of COVID-19 symptoms resolving [21–28]. Such information is vital in treatment planning for long COVID and in providing patients with realistic expectations about their post-acute COVID-19 recovery process. In the acute phase of COVID-19, patients hospitalized for COVID-19 in Zambia had a median recovery time of 12 days [29]. In the post-acute phase, it’s reported that some individuals still had symptoms at nearly two months of follow up [15,27]. None of these studies, however, report on the trajectory or temporal dynamics of long COVID-19 resolution. In this study, we analyzed persistent COVID-19 symptoms to determine the incidence rate, median survival time, factors associated with time-to-resolving, and changes in hazard rate of symptoms resolving.

## Methods

### Study setting

In August 2020, the Zambian Ministry of Health launched 13 referral clinics to provide post-acute COVID-19 (PAC-19) care for individuals recovering from COVID-19. PAC-19 clinics were established to provide comprehensive care given the unknown long-term sequalae of COVID-19 at the time [30]. Individuals were also evaluated for persistent or new symptoms to assess whether these were stable, worsening, and/or new since this could reflect development of late complications of COVID-19. The evaluation was through clinicians’ review of systems using standardized forms.

### Study participants

At the first clinic visit, patients’ demographic and medical history were recorded on standardized paper forms (S1 Appendix) which were routinely abstracted into a REDCap electronic database. Clinicians assessed for current post-COVID symptoms and comorbidities (e.g., hypertension, diabetes, cardiovascular disease, HIV or TB) coupled with physical examination and laboratory investigations. A review of systems included assessment for general, cardiovascular, pulmonary, gastrointestinal, urinary, neurologic, musculoskeletal, ENT (ear, nose and throat), mental health, and dermatologic symptoms.

Patients attended up to five follow-up visits (S2 Appendix) based on an individuals’ parameters and clinicians’ recommendation. Follow-up schedules were individualized for physical exams and, when necessary, reviews were also made via phone calls for some individuals. Discharge criteria from PAC-19 clinics was based on clinicians’ assessment that patients had returned to previous state of health and had normal physical/laboratory parameters. Patients with new medical conditions onset (e.g., hypertension, diabetes mellitus, cardiovascular diseases, stroke, or pulmonary failure) or requiring further specialist care (e.g., physiotherapy, cardiology, endocrinology, nephrology, mental health, and pulmonology) were referred for further follow-up at appropriate units.

### Study design

We implemented a retrospective cohort study design (S1 Fig). Participants were included in the study if they presented in the PAC-19 clinics between 20-Aug-20 (study start date) and 31-Dec-22 (study end date), and had their information captured in a REDCap (Research Electronic Data Capture) electronic database [31]. Data from REDCap was initially accessed on 5-Jan-2024 and analyzed for this study. To ensure the cohort was similar on all baseline characteristics, non-hospitalized patients who attended PAC-19 clinic were excluded from the analysis.

### Study variables

The primary exposure variable was hospitalization with “mild” or with “severe” acute COVID-19 during the study period. Severe acute COVID-19 disease was defined as an episode that required supplemental oxygen therapy or intensive care unit admission or treatment with steroids/or remdesivir. The mild COVID-19 cohort were admitted during acute COVID-19 infection but did not require supplemental oxygen therapy or intensive care unit admission or treatment with steroids/or remdesivir. The outcome of interest was whether the COVID-19 symptoms resolved (coded as 1 if participants experienced symptoms resolution at any time point during the study or 0 if they were unresolved) and the time it took to the event. Participants were categorized as censored if their symptoms didn’t resolve by their last follow-up time point or were lost to follow-up (LTFU) but had at least 1 PAC-19 clinic visit. Time-to-resolution was the time interval, in days, from the date of COVID-19 diagnosis to the date of symptom resolving or censor.

Other study covariates were sex, age group, dominant SARS-CoV-2 variant in Zambia at time of diagnosis, presence of baseline comorbidities, new onsite comorbidities, length of hospitalization, and vaccination status (time-varying covariate). The classification of SARS-CoV-2 dominant variant was based on data from Zambia’s genomic surveillance system submitted to the Global Initiative on Sharing All Influenza Data (GISAID) and previous reports [32,33]. Per GISAID, the wild type variant was dominant from 3-March-2020–21-September-2020, the Beta variant up to 19-March-2021, the Delta variant up to 12-December-2021 and the Omicron variant was still dominant at the study end date of 31-December-2022. Vaccination status was classified as unvaccinated, prior vaccination (defined as 1 or more dose of an approved COVID-19 vaccine) for those vaccinated before their acute COVID-19, and vaccinated during PAC-19 clinic follow up. This was based on vaccination records, when available, or patients’ self-reported status.

### Bias

To control for selection bias, all participants who presented at PAC-19 clinics and met the inclusion criteria were in the study to ensure representativeness of the target population. To minimize measurement bias, patients symptoms were systematically assessed using a standardized review of systems form (S1 Appendix and S2 Appendix). Confounding bias was controlled by including other covariates in addition to the exposure variable at multivariable analysis.

### Sample size

The required minimum sample size was 396 based on the cohort studies sample size formula reported elsewhere, a previously reported long COVID prevalence assumed at 41% among a severe acute COVID-19 cohort and at 17% among mild COVID-19 cohort [34–36]. We further assumed a ratio of severe COVID-19 to mild COVID-19 of two. We nonetheless considered a full enumeration of 823 participants that met the study inclusion criteria for representativeness.

### Statistical analysis

Using descriptive statistics, we assessed baseline demographic and clinical characteristics of study participants. Frequencies with proportions were presented for categorical covariates and medians with the interquartile range (IQR) for non-normally distributed numeric covariates.

At inferential statistics, we fitted the Kaplan-Meier estimator to compute the total person time at risk, symptom resolution incidence rate, and the median survival time. Survival was defined as the probability of not having experienced symptoms resolution up to a certain time. Time was the number of days to symptoms resolution or censor. We assessed factors associated with time-to-resolution of persistent COVID-19 symptoms using a Cox proportional hazard model. In this model, we checked for the proportional hazard assumption by plotting the Schoenfeld residual against the transformed time to check linearity. A Cox proportional hazard diagnostic test was then conducted by computing the correlation between the scaled Schoenfeld residual and time for each covariate to ensure non-significant correlation (p > 0.05). We fitted a stratified Cox proportional hazard model (omnibus p-value = 0.159) to control for violation of proportionality assumption by the covariates presence of comorbidity (p = 0.002) and hospital length of stay (p < 0.001). All covariates in the stratified model had non-significant (p > 0.05) correlation between the scaled Schoenfeld residual and time. Vaccination status was treated as time-dependent covariate to handle its’ significant p-value (S1 Table). In this model, the hazard was defined as the instantaneous risk of experiencing long COVID resolution at a specific moment in time, given that the symptoms had survived up to that time.

Since the data had a longitudinal structure with repeated observation of symptoms over multiple clinical review visits, we adopted a parsimonious approach to fitting a stratified Cox proportional hazard model that accounted for the potential correlation of observations within patients. We first fitted a standard Cox proportional hazard model that did not account for the within-patient dependence of symptoms resolving. This was followed by a model incorporating a sandwich (robust) estimator to adjust for clustering of repeated symptoms observations within patients. The model with clustering were fitted with as stratified time-dependent Cox proportional hazard model at the patient level to capture unobserved heterogeneity due to repeated measurements of COVID-19 symptoms resolving. This model was preferred based on improved fit, as indicated by a higher log-likelihood and the lowest Akaike Information Criteria (AIC) and Bayesian Information Criteria (BIC).

To better understand how the probability of persistent COVID-19 symptoms resolving evolved during follow-up, we fitted a series of parametric survival models that allowed for estimation of the hazard rate – the instantaneous risk of symptoms resolving on a given person-day – to vary according to a specified functional form. This is for the reason that the Kaplan–Meier estimator provides a non-parametric summary of time-to-symptoms resolution by assuming a constant underlying hazard. This approach captured potential changes in the risk of recovery over time, critical periods of symptoms resolution and the time-varying effects of covariates. To determine the functional or parametric form of the underlying hazard rate that symptoms resolving survival time may have followed, five alternative probability distributions (exponential, Weibull, log-logistic, log-normal, and generalized gamma) were evaluated to identify the most appropriate trajectory of persistent symptoms resolving. Model comparison based on log-likelihood and AIC values indicated that the generalized gamma probability distribution best described the data (S2 Fig). Using this model, we estimated and visualized the temporal dynamics of symptom resolution by plotting the evolving hazard rate alongside the cumulative incidence of recovery, illustrating the temporal dynamics of how and when the likelihood of persistent COVID-19 symptoms resolving changed over time. All analysis were done in R version 4.5.1 and statistical significance was considered at p < 0.05.

### Ethics

Ethical clearance (waiver for informed consent) was obtained from the University of Zambia Biomedical Research Ethics Committee (Ref No. 3482-2022), and approval to conduct the study was obtained from the Zambia National Health Research Authority (Ref No: NHRA0000011/09/02/2023). The investigators did not interact with study participants but only had access to anonymized data.

## Results

Of the 823 study participants, slightly over half were female (50.6%) and the median age was 54 (interquartile rang [IQR]: 43–64) years (Table 1). Close to one-third (n = 303, 36.8%) had their symptoms resolved by their first visit to a PAC-19 clinic with reference to the date of COVID-19 diagnosis. Overall, 597 (72.5%) participants had their COVID-19 symptoms resolved during the five follow up visits. Of the total number of participants, 70.1% (501) were diagnosed with COVID-19 when Delta was the dominant variant. Four hundred thirty-seven (57.4%) participants had baseline comorbidities, with the most frequently reported underlying medical conditions being hypertension (40.6%), diabetes mellitus (16.2%), HIV (15.6%) and cardiovascular disease (6.6%). Of all patients, 115 (14%) had new medical conditions onset at the time of acute COVID-19 that included diabetes (n = 59, 51.3%), hypertension (n = 50, 43.5%) and other medical conditions (n = 19, 16.5%).

**Table 1:** Baseline Demographic and clinical characteristics of study participants who presented for care in specialized PAC-19 clinics in Zambia, Aug. 2020 to Dec. 2022 (N = 823).

Characteristic	Longitudinal Follow up Review visits Attendance				
	Visit 1 (823) n (%)	Visit 2 (374) n (%)	Visit 3 (170) n (%)	Visit 4 (83) n (%)	Visit 5 (36) n (%)
**COVID-19 Symptoms Survival status**					
Unresolved	520 (63.2)	218 (58.3)	99 (58.2)	40 (48.2)	12 (33.3)
Resolved	303 (36.8)	156 (41.7)	71 (41.8)	43 (51.8)	24 (66.7)
**Sex**					
Male	400 (49.4)	175 (47.4)	81 (48.5)	41 (49.4)	16 (44.4)
Female	410 (50.6)	194 (52.6)	86 (51.5)	42 (50.6)	20 (55.6)
(Missing)	13	5	3	0	0
**Age (years)**					
Median (IQR)	54 (43, 64)	57 (47, 66)	59 (49, 68)	61 (50, 69)	61 (52, 69)
(Missing)	11	2	1	1	1
**Age group (years)**					
<29	55 (6.8)	9 (2.4)	3 (1.8)	1 (1.2)	1 (2.9)
30-39	89 (11.0)	31 (8.3)	13 (7.7)	4 (4.9)	1 (2.9)
40-49	175 (21.6)	82 (22.0)	29 (17.2)	15 (18.3)	6 (17.1)
50-59	203 (25.0)	97 (26.1)	43 (25.4)	16 (19.5)	5 (14.3)
60+	290 (35.7)	153 (41.1)	81 (47.9)	46 (56.1)	22 (62.9)
(Missing)	11	2	1	1	1
**Dominant SARS-CoV-2 variant at diagnosis** ^**†**^					
Wild Type	39 (5.5)	21 (6.2)	17 (11.0)	7 (8.8)	3 (8.6)
Beta	82 (11.5)	51 (15.1)	26 (16.8)	9 (11.3)	2 (5.7)
Delta	501 (70.1)	249 (73.9)	109 (70.3)	61 (76.3)	30 (85.7)
Omicron	93 (13.0)	16 (4.7)	3 (1.9)	3 (3.8)	0 (0)
(Missing)	108	37	15	3	1
**Presence of comorbidities** ^**‡**^					
No	346 (42.6)	136 (36.6)	52 (30.8)	22 (26.5)	7 (19.4)
Yes	467 (57.4)	236 (63.4)	117 (69.2)	61 (73.5)	29 (80.6)
(Missing)	10	2	1	0	0
**New medical conditions onset ^§^**					
No	708 (86.0)	316 (84.5)	144 (84.7)	69 (83.1)	31 (86.5)
Yes	115 (14.0)	58 (15.5)	26 (15.3)	14 (16.9)	5 (13.5)
**Hospitalization duration (days)**					
Median (IQR)	8 (4, 16)	9 (6, 19)	11 (6, 20)	10 (6, 19)	9 (6, 19)
(Missing)	228	104	43	15	5
**Hospital length of stay (days)**					
1-3	110 (18.5)	26 (9.6)	10 (7.9)	4 (5.9)	2 (6.5)
4-7	162 (27.2)	73 (27.0)	33 (26.0)	19 (27.9)	10 (32.3)
8-14	157 (26.4)	81 (30.0)	39 (30.7)	21 (30.9)	9 (29.0)
≥15	166 (27.9)	90 (33.3)	45 (35.4)	24 (35.3)	10 (32.3)
(Missing)	228	104	43	15	5
**Severe acute COVID-19** ^**¶**^					
No	115 (15.7)	39 (11.3)	17 (10.7)	9 (11.1)	4 (11.4)
Yes	616 (84.3)	307 (88.7)	142 (89.3)	72 (88.9)	31 (88.6)
(Missing)	92	28	11	2	1
**Vaccination status** ^**#**^					
Unvaccinated	625 (83.4)	273 (75.6)	120 (71.9)	44 (53.0)	14 (38.9)
Vaccinated	124 (16.6)	88 (24.4)	47 (28.1)	39 (47.0)	22 (61.1)
(Missing)	74	13	3	0	0
**Time since COVID-19 diagnosis (days)**					
Median (IQR)	30 (20, 42)	51 (37, 70)	75 (57, 99)	92 (74, 115)	110 (97, 143)
(Missing)	109	37	15	3	1

† Dominant SARS CoV-2 variants classification based on GISAID (2023) rather than sequenced specimens from study participants.

‡ Pre-existing comorbidity: hypertension, diabetes, cardiovascular disease, cancer, immunosuppression, chronic lung, kidney, and liver diseases, obesity, HIV and TB.

§ New medical conditions onset included diabetes, hypertension, deep vein thrombosis/pulmonary embolism and kidney injury/disease.

¶ Composite variable for acute COVID-19 episodic that required supplemental oxygen therapy, intensive care unit stay or treatment with steroids/remdesivir.

# Vaccination status was a time vary covariate. Some participants initially unvaccinated got vaccinated during follow up.

During acute COVID-19 hospitalization, 616 (84.3%) participants had severe disease, and the median hospital length of stay was 8 (IQR: 4–16) days. Of all participants, 124 (16.6%) were vaccinated (at least ≥1 dose) prior to COVID-19 infection, however, the number of vaccinated participants more than doubled to 320 (38.9%) by the end of study. Of those vaccinated, 140 (43.8%) received AstraZeneca (AZD1222), 152 (47.5%) Johnson and Johnson’s Janssen (Ad26.COV2.S), 27 (8.4%) did not know the vaccine type they received and 1 (0.3%) received Pfizer-BioNTech (BNT162b2). Vaccines first became available in Zambia in April 2021 before the start of the Delta-dominant phase [33]. A majority of participants (196, 61.3%) got vaccinated during study follow up. The median time since COVID-19 diagnosis participants were followed up was 42 (IQR: 26–72) days while the maximum follow-up time was 260 days.

**Fig 1:**
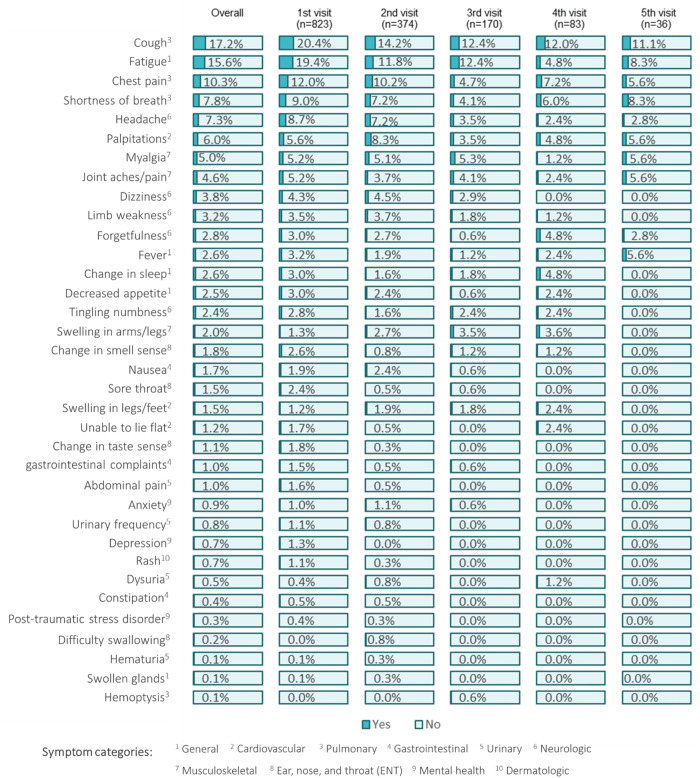
Frequency of long COVID symptoms by PAC-19 clinic visit in Zambia, Aug-20 to Dec-22.

Overall, the most prevalent persistent COVID-19 symptom classes participants presented with included pulmonary (cough 17.2%, chest pain 10.3% and shortness of breath 7.8%), general symptoms (fatigue 15.6%), neurologic (headache 7.3%), cardiovascular (palpitations 6.0%) and musculoskeletal (myalgia 5.0% and joint aches/pain 4.6%) related. The proportions of reported symptoms displayed a declining trend across clinical visits (Fig 1).

**Fig 2:**
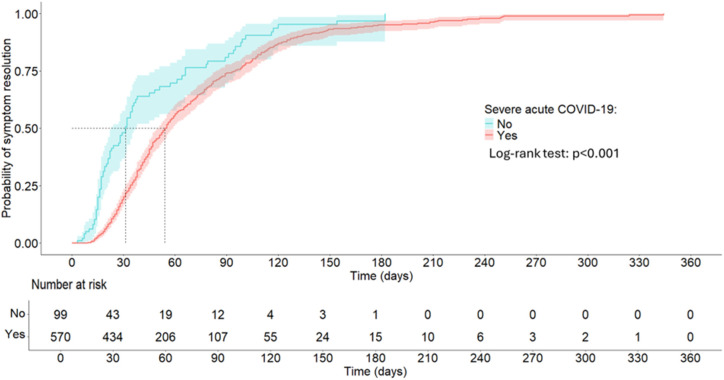
Kaplan-Meier survival curve for post-acute COVID symptoms resolving by severe acute COVID-19 status, Aug-20 to Dec-22.

In this study, the total person-time at risk was 38,162 person-days. Assuming constant risk, the overall incident rate of COVID-19 symptoms resolving was 12.2 per 1,000 person-days. The resolution incident rate was higher in the mild acute COVID-19 cohort compared to the severe COVID-19, 17.8 vs 11.6 per 1,000 person-days, respectively. In severe acute COVID-19 cohort, the median survival time of symptoms was 54 (IQR: 37–107) person-days while it was 31 (IQR: 17–89) person-days in the mild acute COVID-19 cohort (Fig 2).

In both unadjusted and adjusted survival analysis of persistent COVID-19 symptoms, the stratified Cox proportional hazard model – which accounted for clustering of participants within PAC-19 clinics – showed a better overall fit than the standard model. The model fit indices were lower, with a log-likelihood of –1,547.073, an AIC of 3,116.145, and a BIC of 3,161.420, compared to higher model indices for the standard model (Table 2). This model was preferred based on improved overall fit.

**Table 2:** Factors associated with time-to-resolution of persistent COVID-19 symptoms in Zambia, Aug-20 to Dec-22.

	Stratified Cox Proportional Hazard Cluster† Model			
Variables	Unadjusted HR (95% CI)	p-value*	Adjusted HR (95% CI)	p-value*
**Sex**				
Male	*Referent*	–	*Referent*	–
Female	0.940 (0.79-1.12)	0.498	0.90 (0.73-1.11)	0.339
**Age group (years)**				
≤29	*Referent*	–	*Referent*	–
30-39	0.69 (0.40-1.21)	0.103	0.96 (0.56-1.65)	0.882
40-49	0.74 (0.46-1.19)	0.125	1.05 (0.65-1.70)	0.831
50-59	0.83 (0.52-1.34)	0.239	1.11 (0.96-1.78)	0.676
60+	0.71 (0.32-0.92)	**0.048**	1.03 (0.64-1.65)	0.898
**Dominant SARS-CoV-2 variant at diagnosis** ^‡^				
Wild Type	*Referent*	–	*Referent*	–
Beta	1.12 (0.73-1.72)	0.600	1.35 (0.74-2.47)	0.323
Delta	1.03 (0.72-1.46)	0.883	1.54 (0.90-2.62)	0.113
Omicron	2.32 (1.45-3.71)	**<0.001**	2.71 (1.46-5.03)	**0.002**
**Presence of comorbidities** ^§,¶^				
No	*Referent*	–	*–*	–
Yes	0.61 (0.49-0.76)	**<0.001**	–	–
**Hospital length of stay (days)** ^¶^				
1-3	*Referent*	–	*Referent*	–
4-7	0.71 (0.48-1.05)	0.085	–	–
8-14	0.61 (0.42-0.89)	**0.010**	–	–
≥15	0.35 (0.25-0.51)	**<0.001**	–	–
**Severe acute COVID-19** ^#^				
No	*Referent*		*Referent*	–
Yes	0.56 (0.41-0.76)	<**0.001**	0.68 (0.50-0.92)	**0.026**
**Vaccination status** **				
Unvaccinated	*Referent*		*Referent*	–
Vaccinated	0.83 (0.68-1.00)	0.083	0.79 (0.63-0.98)	**0.038**
	**Description**	**Log-likelihood**	**AIC**	**BIC**
	Standard Stratified Cox proportional hazard model	-1,852.296	3,726.592	3,771.867
	Stratified Cox Proportional Hazard Cluster model	-1,487.343	2994.868	3035.645

**NOTES**

Abbreviation: **HR** - Hazard ratio and **CI** – confidence interval.

* Bolded p-values are significant at p < 0.05.

† Model selection criteria (fit statistics).

‡ Dominant SARS CoV-2 variants classification based on GISAID (2023) rather than sequenced specimens from study patients.

§ Pre-existing comorbidity: hypertension, diabetes, cardiovascular disease, cancer, immunosuppression, chronic lung, kidney, and liver diseases, obesity, HIV and TB.

¶ Stratifying variable for the stratified Cox Proportional Hazard model.

# Composite variable for acute COVID-19 episodic that required supplemental oxygen therapy, intensive care unit stay or treatment with steroids/remdesivir.

** Vaccination status was a time-varying covariate, i.e., a proportion of patients initially not vaccinated got vaccinated during follow up time.

According to the Cox proportional hazard model (Table 2), participants with severe acute COVID-19 disease had a significantly lower likelihood of resolving persistent symptoms (adjusted hazard ratio [aHR]: 0.68; 95% confidence interval [CI]: 0.50-0.92). Those diagnosed during the Omicron-dominant phase had 2.71 times higher hazard of symptom resolution compared to those diagnosed during the wild type dominant phase. Similarly, participants diagnosed during the Beta- (aHR: 1.35) and delta-dominant (aHR: 1.83) phases had increased likelihood of having their symptoms resolved. These effects were, however, non-significant. Vaccination against COVD-19 was associated a reduced likelihood of persistent COVID-19 symptoms resolving (aHR: 0.79; 95% CI: 0.63-0.98). Sex (females vs. males, p = 0.338), and age group (p > 0.05) were not significantly associated with persistent COVID-19 symptoms resolution.

**Fig 3:**
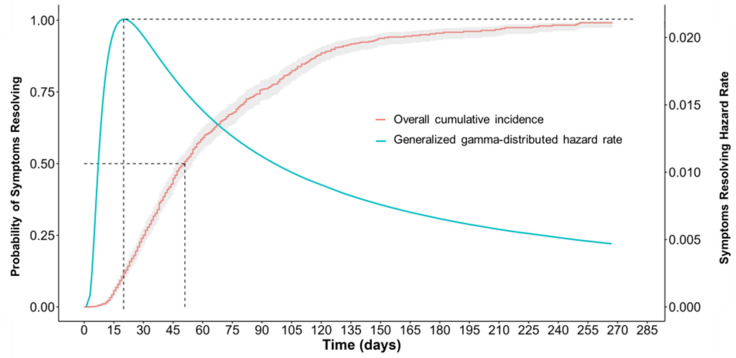
Kaplan-Meier cumulative incidence and generalized gamma-distributed hazard rate for persistent COVID-19 symptom resolving, Aug-2020 to Dec-2022.

At estimation of the underlying hazard rate, the temporal dynamics of persistent COVID-19 symptoms resolving were found to be best characterized by a generalized gamma probability distribution. The functional form of this hazard rate was not constant over time but reflected a non-monotonic recovery pattern with an initial increase, reaching a peak around person-day 20, followed by a gradual decline thereafter (Fig 3). At peak hazard rate, participants had an estimated 2.14% instantaneous probability of persistent COVID-19 symptoms resolving in the next person-day interval, assuming they had not yet resolved. In comparison, the Kaplan-Meier estimator, which assumes a constant underlying hazard and does not allow for adjustment for covariates, indicated a much higher probability (cumulative incidence) of symptoms resolving of 11.7% (95% CI: 9.3-14.1%) at person-day 20. The median survival time from the cumulative incidence Kaplan-Meier curve occurred at person-day 51 (95% CI: 47–55), a month after peak hazard rate when participants had the highest instantaneous probability that symptoms resolution would occur in the next person-day interval.

## Discussion

Factors associated with the risk of COVID-19 symptoms resolving included severe acute COVID-19, diagnosis with SAR-CoV-2 infection during an Omicron variant dominant period and being vaccinated against COVID-19 similar to findings in previous studies [37–41]. Exposure to severe acute COVID-19 and vaccination against COVID-19 were associated with reduced risk of symptoms resolving. Diagnosis during an Omicron variant dominant period was associated with increased risk of symptom resolution. The functional form the underlying hazard rate to symptom resolution was not constant over time but reflected a non-monotonic recovery pattern with an initial increase to peak rate followed by a gradual decline thereafter. The peak rate occurred at under one month and the median survival time occurred a month later. The changes in hazard rate over time is consistent with a report on the survival time dynamics of long-lasting symptoms after acute COVID-19 [42].

The median survival time in this study is consistent with findings from other studies in Zambia [15,27,29]. This may suggest that prolonged illness due to COVID-19 may lead to continued demand for healthcare services even after the acute phase of SARS-CoV-2 infection. In this study, up to half of the patients at the PAC-19 clinic continued to experience persistent symptoms beyond two months and still required medical attention.. Although less than 1% (1,238) of the over 349,000 confirmed COVID-19 cases in Zambia were seen at PAC-19 clinics, a larger proportion of patients likely experienced persistent symptoms that may have contributed to the overall strain on the healthcare system. This highlights the importance of specialized post-acute COVID-19 clinics in providing ongoing care and support for long-term recovery.

The incident rate of symptoms resolution in this cohort was lower than was found in other acute COVID-19 studies [29,43,44]. A lower symptoms resolution incident rate may be related to tissue injury that potentially leads to long-term organ dysfunction and a longer recovery process [3]. Inflammation and immune dysregulation, which is also common in SAR-CoV-2 infections, may have also prolonged recovery and thus a lower symptoms resolution incident rate [45]. Furthermore, post-acute COVID-19 symptoms are multi-system conditions compared to acute COVID-19 symptoms thus they have long-lasting health effects that take more time to resolve.

Severe acute COVID-19 was associated with greater persistent symptoms than mild COVID-19 [46]. This is thought not only to be related to the inflammation of the central nervous system but to intensive medical treatments in severe COVID cases which in turn potentially affect the immune response and the body’s ability to recover [47,48]. Persistent symptoms may furthermore lead to psychological symptoms such as anxiety, depression, and post-traumatic stress disorder which are reported in long COVID patients [49,50]. These may have adversely contributed to the overall ability of the body to recover.

In this study, vaccination was found to be associated with reduced hazard of symptoms resolving consistent with another study on prolonged symptoms in people that receive COVID-19 vaccines when already infected with a SARS-Cov-2 virus [51]. This may be related to the overlap of immune processes, potentially leading to immune dysregulation or complications in symptom recovery [52]. In our study, only about one-sixth of the participants were vaccinated prior to SARS-CoV-2 infection while the majority got their vaccines when already infected with COVID-19. A previous studies have found that receiving COVID-19 vaccines prior to SARS-CoV-2 infection is known to enhance vaccine effectiveness by priming the immune system and allowing for a more effective response [53–55]. This thus highlights the importance of timing COVID-19 vaccines primarily way before a SARS-CoV-2 infection to accord the body time to mount an optimal immune response.

The findings that patients diagnosed with COVID-19 during an Omicron variant dominant period had an increased risk of symptom resolution is consistent with reports that Omicron was less severe than previous lineages [41]. This may be due to immunity occasioned from exposure to previous SARS-CoV-2 variants or vaccination which may have moderated severity of post-acute COVID symptoms [56]. Because of an altered spike protein, the Omicron variant replicates more efficiently in the upper respiratory tract rather than in lungs where more severe respiratory illnesses occur [57]. This may have resulted in less severe lung damage with Omicron compared to other variants and a relatively quicker symptoms resolution.

An increasing then decreasing underlying hazard rate may mean that participants had a higher likelihood of recovering from post-acute COVID-19 symptoms relatively early. This is consistent with what is reported about recovery from COVID-19 in previous studies [15,27,29]. A decreasing hazard rate following a peak rate at 20 person-days, however, may suggest prolonged recovery period for the majority of participants since median survival time occurred one month after peak hazard rate. This information likely may help in treatment planning of long COVID and provide patients with realistic expectations about the timing, likelihood, and patterns of recovery from post-acute COVID-19 symptoms that last more than a month.

The findings in this study are subject to some limitations. Firstly, only patients who were hospitalized during acute COVID-19 and attended post-acute COVID-19 follow up care were included in this study. This may represent some potential limitation as the findings may not be generalizable to patients who were outpatients during acute COVID-19, sought care elsewhere for post-acute COVID-19 management, or did not seek care at all. Post-COVID-19 services were relatively limited in Zambia during this project, and many more people were infected and hospitalized with COVID-19 than the number who attended post-COVID-19 clinics. Secondly, patients’ classification of the dominant SARS-CoV-2 variant at time of diagnosis was not based on sequenced specimen. Although the dominant SARS-CoV-2 variants may have accounted for a majority of new COVID-19 cases at the time of diagnosis, other variants were still circulating thereby presenting potential for variant misclassification. Thirdly, information on prior COVID-19 infection or reinfection was not collected. This limits the ability to differentiate between ongoing symptoms from those resulting from a potential reinfection, particularly beyond 90 days from the original diagnosis when reinfection is more likely to occur.

## Conclusion

Our study found that persistent COVID-19 symptoms in Zambia had a median survival time of about two months and the peak hazard rate at under a month. The median survival time occurring nearly a month after peak hazard rate may suggest that patients that did not initially improve in the first month of their post-acute infection might have a prolonged recovery period. The time to symptoms resolving was, however, associated with severe acute COVID-19, the dominant SARS-CoV-2 variant at diagnosis, prior vaccination status, the presence of comorbidities and hospital length of stay. These results underscore the complexity and dynamics of the time to persistent COVID-19 symptoms resolving and may likely help in treatment planning and providing patients with realistic expectations about their recovery process.

## Supporting information

S1 Fig Study analysis flow diagram of participants presenting in PAC-19 clinic in Zambia, Aug. 2020 to Dec. 2022.
(TIF)

S2 Fig Parametric probability distributions comparison of estimated underlying hazard rate for time to persistent COVID-19 symptoms resolving. (TIF)

S1 Table Model fit statistic and selection criteria. (DOCX)

S1 Appendix Patients’ baseline demographic and medical history standardized forms. (PDF)

S2 Appendix Patients’ Follow-up clinical visit standardized forms. (PDF)
