# Supplementary figures and images for: Parametric survival analysis of long COVID among hospitalized patients in Zambia: A retrospective cohort study on the time to symptoms resolving
Source: PLOS Glob Public Health. 2025 Nov 6;5(11):e0004679. doi: 10.1371/journal.pgph.0004679 (PMC12591408; doi:10.1371/journal.pgph.0004679)

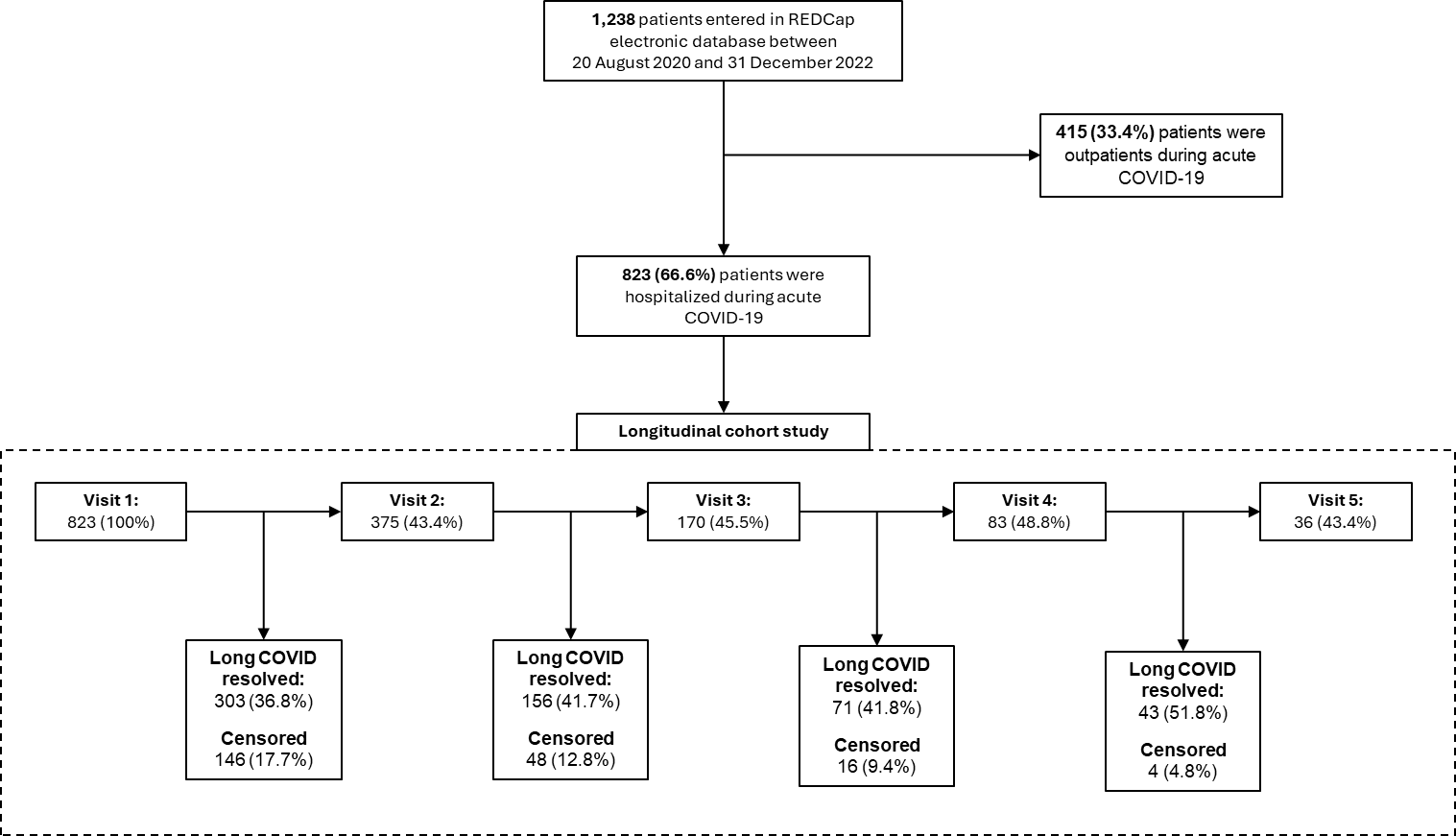

Supplement: S1 Fig — 2020 to Dec. 2022. (TIF) [file pgph.0004679.s001.tif]

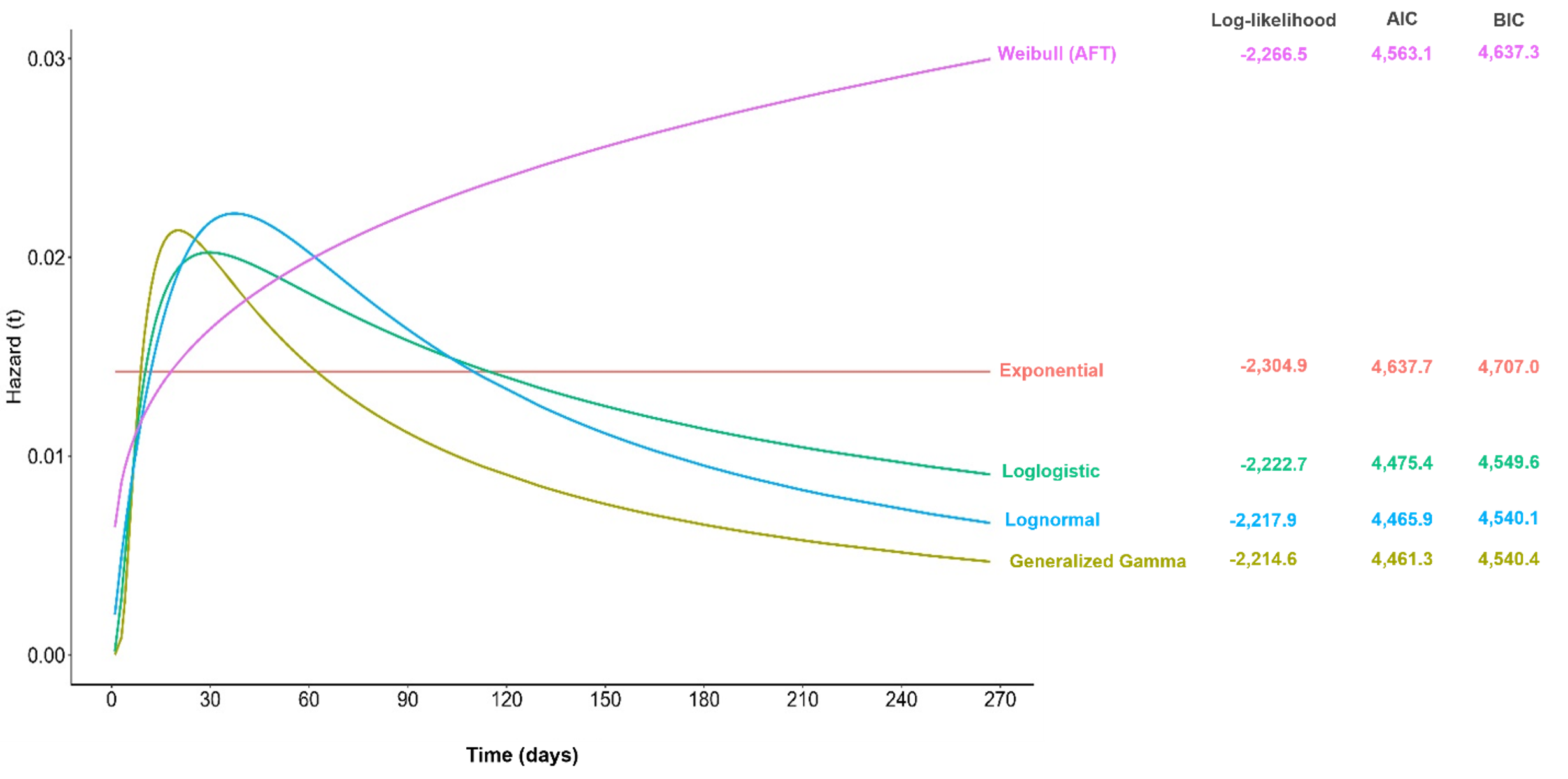

Supplement: S2 Fig — (TIF) [file pgph.0004679.s002.tif]
