# Supplementary material for: Parametric survival analysis of long COVID among hospitalized patients in Zambia: A retrospective cohort study on the time to symptoms resolving
Source: PLOS Glob Public Health. 2025 Nov 6;5(11):e0004679. doi: 10.1371/journal.pgph.0004679 (PMC12591408; doi:10.1371/journal.pgph.0004679)
Supplement: S1 Table — (DOCX) [file pgph.0004679.s003.docx]

**Check for proportional hazards assumption**

| **Covariate** | **p-value*** |
| --- | --- |
| Sex | 0.807 |
| Age group (years) | 0.979 |
| Dominant SARS-CoV-2 variant at diagnosis | 0.202 |
| Presence of comorbidities ^†^ | - |
| Hospital length of stay (days) ^†^ | - |
| Severe acute COVID-19 | 0.052 |
| Vaccination status | **0.036** |
| Omnibus (Global) | 0.159 |
| - Bolded p-values are significant at p<0.05 and were treated as time-varying covariate   ^†^ Stratifying variable for the stratified Cox Proportional Hazard model | |
