# Supplementary material for: Parametric survival analysis of long COVID among hospitalized patients in Zambia: A retrospective cohort study on the time to symptoms resolving
Source: PLOS Glob Public Health. 2025 Nov 6;5(11):e0004679. doi: 10.1371/journal.pgph.0004679 (PMC12591408; doi:10.1371/journal.pgph.0004679)
Supplement: S1 Appendix — (PDF) [file pgph.0004679.s004.pdf]

Post-Covid Condition Baseline Chart

PAC19 ID*: ______________

Date*: ________________________________

Referred from*: ☐ Hospital ☐ HBC ☐ Other

(All fields are required)*

Type of review* ☐ Telephonic ☐ In person

Surname:____
Name: ____
Sex: ☐ Male ☐ Female Age: ______ DOB: ________
Phone number: __________________________________
Address: ______________ District: ________________

Past Medical History*

☐ Hypertension
☐ Cardiovascular disease
☐ Diabetes
☐ Cancer
☐ Immunosuppression
☐ Chronic lung disease
☐ Chronic kidney disease
☐ Chronic liver disease
☐ Obesity
☐ Pregnant

☐ HIV
Recent VL: _____
ART: ☐TLD ☐TLE
☐ Other: _____ ☐None
☐ Tuberculosis
☐ Other PMH:
1) __________
2) __________

*Mode of diagnosis**: ☐ PCR ☐RDT☐Ab☐ CT/CXR ☐symptoms
Date*: ______________
Repeat test : ☐ PCR; Date: ______________
Test result: ☐ Positive ☐ Negative ☐ Don't know
Hospitalized*: ☐ Yes ☐ No Facility name: ______________
Dates of hospitalization*: ____________ to _______________
Required oxygen*: ☐ Yes ☐ No
Type of Care*: ICU ☐ ☐ Non ICU
Meds received: ☐ Steroids ☐ Remdesivir ☐ anticoagulant
☐ Colchicine ☐Antibiotics Other ☐_______________
Newly diagnosed conditions*: ☐ Diabetes ☐ Hypertension
☐ DVT/PE ☐ Kidney injury/disease ☐Other: ___________
Vaccinated*: ☐ Yes ☐No Type: _______________________________
Dose 1 ☐ Date*: ______________
Dose 2 ☐ Date*: ______________
Notes:____
____
____

Current Medications and Allergies

1) ___________________
2) ___________________
3) ___________________
4) ___________________
5) ___________________
6) ___________________
7) ___________________
8) ___________________
9) ___________________
10)___________________

Drug allergies: _________

☐ None

☐ Current TB* *TB meds*: ____________________
☐ presumptive TB (proceed with screening)*

Social History

Employed ☐ Yes ☐ No Job: ____________________
Number persons in household: ____________________
☐ Smoke cigarettes ☐ Drink alcohol ☐ Use drugs

Symptoms*

Cough	☐ Yes ☐ No
Fever	☐ Yes ☐ No
Night sweats	☐ Yes ☐ No
Weight loss	☐ Yes ☐ No
Chest pain	☐ Yes ☐ No

If *Yes* to any of the above, test the patient for tuberculosis*

Functional Status Since Covid-19 (*Over Past 7 Days*)*

	Same	Better	Worse
Ability to self-care	☐	☐	☐
Taking care of household tasks	☐	☐	☐
Your day-to-day work/school	☐	☐	☐
Standing for > 30 minutes	☐	☐	☐
Walking long distances (>1km)	☐	☐	☐
Washing body	☐	☐	☐
Getting dressed	☐	☐	☐
Concentrating for ten minutes	☐	☐	☐
Mood	☐	☐	☐
Sleep	☐	☐	☐

Review of Systems (*Currently*)*

General
☐ Fever
☐ Fatigue
☐ Decreased appetite
☐ Swollen glands
☐ Change in sleep

Cardiovascular
☐ Unable to lie flat
☐ Palpitations
☐ Swelling in legs/feet

Pulmonary
☐ Cough
☐ Shortness of breath
☐ Chest pain
☐ Hemoptysis

Gastrointestinal
☐ Nausea
☐ Abdominal pain
☐ Diarrhea
☐ Constipation

Urinary

Neurologic
☐ Headache
☐ Forgetfulness
☐ Dizziness
☐ Limb weakness
☐ Tingling/numbness

Musculoskeletal
☐ Joint aches/pain
☐ Muscle aches/pain
☐ Swelling in arms/legs

ENT
☐ Sore throat
☐ Change in taste sense
☐ Change in smell sense
☐ Difficulty swallowing

PSYCHIATRIC (fill after mental health screening tools)
☐ Depression
☐ Anxiety
☐ PTSD

	☐ Frequency<br>☐ Dysuria<br>☐ Hematuria	*Dermatologic*<br>☐ Rash<br>Other symptoms<br>☐ ______________<br>☐ ______________

PhysicalExam

Height: ______ Weight: _______ BMI: _______
Temp: _______ HR: _______ BP: _______ RR: ______
O2 Saturation*: _________ FBS: _______ RBS: ______

	Skin	ENT	Heart	Lungs	Abd	MSS	Neuro
Normal	☐	☐	☐	☐	☐	☐	☐
Abnormal	☐	☐	☐	☐	☐	☐	☐

Notes: ____
____

TB Testing*

Was person tested for TB?	☐ Yes ☐ No
Date of test	dd/mm/yyyy
Type of test	☐ Xpert ☐ uLAM ☐ CXR ☐ Smear/culture
Is result available yet?	☐ Yes ☐ No
Result	☐ Positive ☐ Negative ☐ Indeterminate ☐ Unknown

Notes

Labs and Studies

Laboratory test			radiology	
tests	dates	results	dates	reports
Hb				CXR:
WCC				
MCV				
Plts				
Neu				ECG:
Lym				
Potassium				
Urea				
Creatinine				CT scan:
Sodium				
AST				
ALT				
INR				Echocardiogram:
D-dimer*				
CRP*				
LDH				
CK				Others:
Ferritin				
HbA1C				
TB				
Others				

Assessment*	Plan*
1____________________________________<br>____________________________________<br>2____________________________________<br>____________________________________<br>3____________________________________<br>____________________________________<br>4____________________________________<br>____________________________________<br>5 ____________________________________<br>____________________________________	1-Tests:<br><br>2- Prescriptions:<br><br>3-Referral* ☐ Endocrinologis ☐ Psychiatry ☐ Cardiologist<br>☐ Nephrologist ☐ lmonologist ☐ physiotherapy<br>☐ others ____________________________

**Has the patient returned to previous state of health?** **Yes** ☐ **No** ☐	
	4-Next review*: ☐ Tele ☐ In person<br><br>Date: _______________<br><br>5. Discharge Date(**if discharged):*** _______________________
