# Supplementary material for: Parametric survival analysis of long COVID among hospitalized patients in Zambia: A retrospective cohort study on the time to symptoms resolving
Source: PLOS Glob Public Health. 2025 Nov 6;5(11):e0004679. doi: 10.1371/journal.pgph.0004679 (PMC12591408; doi:10.1371/journal.pgph.0004679)
Supplement: S2 Appendix — (PDF) [file pgph.0004679.s005.pdf]

Post-Covid Condition Follow up Chart	PAC19 ID*: ______________

Surname*: ____

Name*: ____

Date*: _____________________________

✧ **Telephonic review**

Vaccinated*: ☐ Yes ☐ No Type: ________ Dose 1 ☐ Date: ______________ Dose 2 ☐ Date: ______________

Assessments*:____

____

____

____

plan:____

____

____

Next review*: ☐ Tele ☐ in person

Date: ______________

✧ **In person review**

Vaccinated*: ☐ Yes ☐ No Type: ________ Dose 1 ☐ Date: ______________ Dose 2 ☐ Date: ______________

Height: ______ Weight: ______ BMI: ______
Temp: _______ HR: _______ BP: _______ RR: ______O2 Saturation*: _________ FBS: _______ RBS: ______

*General**
- ☐ Fever
- ☐ Fatigue
- ☐ Decreased appetite
- ☐ Swollen glands
- ☐ Change in sleep

Cardiovascular
- ☐ Unable to lie flat
- ☐ Palpitations
- ☐ Swelling in legs/feet

*Pulmonary**
- ☐ Cough
- ☐ Shortness of breath
- ☐ Chest pain
- ☐ Hemoptysis

*Gastrointestinal**
- ☐ Nausea
- ☐ Abdominal pain
- ☐ Diarrhea
- ☐ Constipation

*Urinary**
- ☐ Frequency
- ☐ Dysuria
- ☐ Hematuria

*Neurologic**
- ☐ Headache
- ☐ Forgetfulness
- ☐ Dizziness
- ☐ Limb weakness
- ☐ Tingling/numbness

Musculoskeletal
- ☐ Joint aches/pain
- ☐ Muscle aches/pain
- ☐ Swelling in arms/legs

*ENT**
- ☐ Sore throat
- ☐ Change in taste sense
- ☐ Change in smell sense
- ☐ Difficulty swallowing

*PSYHIATRIC (fill after mental health screening tools)**
- ☐ Depression
- ☐ Anxiety
- ☐ PTSD

Dermatologic
- ☐ Rash

Other symptoms*
- ☐ ____________
- ☐ ____________

Laboratory test			Radiology	
tests	dates	results	dates	reports
Hb				CXR:
WCC				
MCV				
Plts				
Neu				ECG:
Lym				
Potassium				
Urea				
Creatinine				CT scan:
Sodium				
AST				
ALT				
INR				Echocardiogram:
D-dimer*				
CRP*				
LDH				
CK				Others:
Ferritin				
HbA1C				
Others				

	Skin	ENT	Heart	Lungs	Abd	MSS	Neuro
Normal	☐	☐	☐	☐	☐	☐	☐
Abnormal	☐	☐	☐	☐	☐	☐	☐

Notes:

____

____

____

____

Follow up on Tuberculosis screening*

Was person tested for TB?	◻ Yes ◻ No
Date of test	dd/mm/yyyy
Type of test	◻ Xpert ◻ uLAM ◻ CXR ◻ Smear/culture
Is result available yet?	◻ Yes ◻ No
Result	◻ Positive ◻ Negative ◻ Indeterminate ◻ Unknown

Notes: ____
____
____

Assessment*	Plan*
1____<br>____<br>____	1-Tests:
2____<br>____<br>____	2- Prescriptions:
3____<br>____<br>____	3-Referal*: ☐ Endocrinologist ☐ Psychiatry ☐ Cardiologist ☐ Nephrologist ☐ Pulmonologist ☐ Physiotherapy others ☐
4____<br>____<br>____<br><br>**Has the patient returned to previous state of health? * Yes** ☐ **No** ☐	4-Next review* ☐ Tele ☐ in person<br>Date: _______________<br><br>5. Discharge Date***(if patient is discharged)****________________
